# Why Have the Benefits of DHA Not Been Borne Out in the Treatment and Prevention of Alzheimer’s Disease? A Narrative Review Focused on DHA Metabolism and Adipose Tissue

**DOI:** 10.3390/ijms222111826

**Published:** 2021-10-31

**Authors:** Rory J. Heath, Thomas R. Wood

**Affiliations:** 1 Emergency Medicine Department, Derriford Hospital, University Hospitals Plymouth, Plymouth PL6 8DH, UK; rory.heath2@nhs.net; 2 Department of Pediatrics, University of Washington, Seattle, WA 98195, USA; 3 Center on Human Development and Disability, University of Washington, Seattle, WA 98195, USA; 4 Institute for Human and Machine Cognition, Pensacola, FL 32502, USA

**Keywords:** DHA, omega-3, Alzheimer’s, dementia, neurodegeneration

## Abstract

Docosahexaenoic acid (DHA), an omega-3 fatty acid rich in seafood, is linked to Alzheimer’s Disease via strong epidemiological and pre-clinical evidence, yet fish oil or other DHA supplementation has not consistently shown benefit to the prevention or treatment of Alzheimer’s Disease. Furthermore, autopsy studies of Alzheimer’s Disease brain show variable DHA status, demonstrating that the relationship between DHA and neurodegeneration is complex and not fully understood. Recently, it has been suggested that the forms of DHA in the diet and plasma have specific metabolic fates that may affect brain uptake; however, the effect of DHA form on brain uptake is less pronounced in studies of longer duration. One major confounder of studies relating dietary DHA and Alzheimer’s Disease may be that adipose tissue acts as a long-term depot of DHA for the brain, but this is poorly understood in the context of neurodegeneration. Future work is required to develop biomarkers of brain DHA and better understand DHA-based therapies in the setting of altered brain DHA uptake to help determine whether brain DHA should remain an important target in the prevention of Alzheimer’s Disease.

## 1. Introduction

Alzheimer’s Disease (AD) is the most common cause of dementia [1]. Its prevalence is predicted to continue to increase, potentially affecting up to 14 million individuals by 2050 in the US alone [2]. No cure for AD yet exists; however, efforts to prevent AD have identified modifiable risk factors such as diet and lifestyle [3,4].

Docosahexaenoic acid (DHA) is a N3 long-chain polyunsaturated fatty acid (LCPUFA) found in abundance in fish and other seafoods [5]. DHA synthesis from other N3 polyunsaturated fatty acid (PUFA) precursors occurs inefficiently in the liver, with some variation determined by genetic polymorphism [6,7,8], and optimal DHA levels are largely thought to require some dietary DHA [9,10,11]. Furthermore, DHA is essential for proper neurological development and functioning throughout the lifespan. The brain relies on DHA supplied via plasma, and deficiency impairs proper development of vision, cognitive functioning, and behavior [12,13,14,15,16].

Intake of dietary fish and other seafoods, which are rich sources of DHA, is associated with reduced risk of AD in populations of multiple countries [17,18,19,20]. A meta-analysis by Zhang et al. concluded that increases in daily DHA intake by 0.1 g/day reduced risk of AD by 37% [21]. In the laboratory, DHA has direct effects against multiple pathological hallmarks of AD such as neuroinflammation, amyloid beta, and tau protein neurofibrillary tangles, and influences the cellular response to inflammation and injury [22,23,24,25,26]. DHA-derived metabolites termed specialized pro-resolving mediators (SPMs) also act to inhibit inflammation and apoptosis and to promote pro-survival cellular signaling after injury [27,28,29].

Despite the myriad influences of DHA on AD neuropathological mechanisms, and the association of greater fish intake with a reduced risk of AD at an epidemiological level, manipulation of dietary DHA intake by fish oil supplementation has had little consistent effect to prevent or ameliorate AD [30]. More recently, the understanding that different dietary and plasma DHA forms have specific metabolic fates affecting distribution into tissues may help to explain why fish oil supplementation does not provide the beneficial effects expected from pre-clinical and epidemiological evidence. It has been argued that the brain has limited uptake of triglyceride (TG)-DHA found in fish oil supplements, instead favoring DHA in lysophospholipid forms, such as lysophosphatidylcholine (LPC), derived from dietary phospholipid (PL) [31,32,33,34]. Lysophosphatidylcholine-DHA (LPC-DHA) enters the brain via the Mfsd2a transporter, a selective transporter of lysophospholipid-bound fatty acids [35].

This paper begins by outlining the ways in which DHA may contribute to protection against AD, followed by a discussion of the evidence supporting the concept of specific metabolic fates of dietary DHA sources and forms with respect to distribution to tissues including brain. Specific metabolic fates of DHA are well represented in short-duration studies following acute administration, emphasizing the preferential uptake of phospholipid and lysophospholipid forms of DHA into brain, a phenomenon which may provide future therapeutic benefit to states of altered DHA brain uptake [36]. Studies of increasing duration, however, show that the effects of dietary DHA form upon tissue distribution become less significant, and instead suggest that plasma DHA as a non-esterified fatty acid (NEFA) is the main supplier of brain DHA requirements.

The reduced dependence on dietary form with time suggests the presence of other factors affecting plasma DHA homeostasis. Dietary TGs are metabolically fated to deposit in adipose tissue, an organ that can provide a rich and constant source of DHA to tissues such as brain that outweighs dietary DHA in terms of mass and reliability of supply. We therefore discuss the significant role of adipose tissue, rather than nuances of dietary form, as a major unexplored factor affecting DHA homeostasis in the body and brain in health and disease. We also outline the importance of future work to develop better biomarkers of brain DHA, and better understand DHA uptake into the brain in diseased states to help determine whether brain DHA should remain a specific target in the prevention and treatment of AD.

## 2. DHA Benefits Alzheimer’s Disease Neuropathological Mechanisms

Mechanistic studies from molecular, cellular, and in vivo models have shown the beneficial effects of DHA against key neuropathological processes associated with AD development and pathophysiology.

### 2.1. Amyloid, Tau, Neuroinflammation and Cell Signaling

AD is characterized by neurofibrillary tangles (NFT) of tau protein within neurons, deposition of amyloid beta plaques throughout the brain, and synaptic loss [37]. DHA may have direct effects against amyloid beta in vitro and in vivo [22,38,39], and may reduce intraneuronal formation of tau [25]. DHA is also integral to membrane fluidity and permeability, thus DHA deficiency can alter the function of membrane-bound enzymes and trans-membrane transport abilities, affecting neuronal signaling and learning ability [24,40,41,42]. DHA influences cell fate signaling, promoting neuronal differentiation and neurogenesis of hippocampal neurons [43], loss of which is seen in AD [44]. Furthermore, DHA improves glutathione and antioxidant status, inhibits mediators of apoptosis [23,45,46], and modulates cell death and injury size after hypoxic-ischemic injury [47,48].

AD pathogenesis is also associated with chronic neuroinflammation [49]. DHA and its downstream metabolites, which form a subset of ‘Specialized Pro-resolving Mediators’ (SPMs), act to inhibit inflammatory signaling, inhibit amyloid-beta associated apoptosis and promote anti-apoptotic gene expression [50], to foster the return of injured tissues to pre-injury homeostasis [27,51,52]. The prototypical SPM of the central nervous system, neuroprotectin D1 (NPD1) is produced via lipoxygenation of DHA after oxidative insults and ischemia-related injury [53], and induces pro-survival and anti-inflammatory signaling [54]. Amyloid-beta induces formation of NPD1 from DHA, while NPD1 protects against amyloid-beta induced cytotoxic apoptosis [50]. Similarly, the arachidonic acid-derived SPM Lipoxin A4 (LXA4) protectively modulates microglia activation in mouse models of AD [55]. NPD1 is decreased in AD hippocampus [56], and LXA4 is decreased in AD cerebrospinal fluid and brain [29], suggesting alteration of the resolution of neuroinflammation as a key event in AD development that downstream metabolites of DHA may actively contribute to.

### 2.2. DHA Improves Vascular Risk Factors for Dementia

DHA acts at the intersection of cardiovascular disease and AD, pathologies that often co-exist in older persons [4]. DHA supplementation benefits metrics of cardiovascular health such as total serum TGs and measures of arterial stiffness [57]. Higher dietary N3 PUFA intake has been associated with fewer cerebral infarcts [58] as well as a reduction in the risk of age-related decline in verbal fluency, with particular benefit to those with hypertension [55]. DHA therefore has wide peripheral effects that may also indirectly reduce the risk of neurodegenerative disease.

## 3. DHA Status of Peripheral Biomarkers Correlates with AD Risk and Progression

Considering the multiple pathways through which DHA may reduce the risk of AD, an accurate measure of brain DHA status would be critical to both assess patient risk and track response in clinical trials. In vivo imaging techniques have shown widespread alteration of phospholipid composition in key areas of AD brain such as in hippocampus [59,60]. Autopsy studies have shown worsened loss of phospholipid content in frontal, temporal and hippocampal brain regions in individuals developing AD below the age of 65 in comparison to those developing ‘senile dementia’ in later life, suggesting that alterations in brain fatty acid composition are linked with worsened neurodegeneration [61]. In particular, the content of the phospholipid phosphatidylethanolamine (PE) of AD brains has been reported to be reduced in comparison to controls [62,63,64,65,66,67].

With regard to the DHA content of AD brain, PE-bound DHA was found to be reduced in the hippocampi and parahippocampi by up to 45% by Prasad et al. and up to 15% by Guan et al. [68,69] Furthermore, in samples of frontal cortex, Nakada et al. found reductions in PE-DHA in AD, as well as reductions in AD content of two more of the four main phospholipids, phosphatidylcholine (PC) and phosphatidylinositol (PI) [70]. Lukiw et al. found DHA levels half those of normal controls in the hippocampus [50]. Other studies have shown no alteration of DHA in AD brain [71,72], including the largest study of autopsied AD brains [73]. However, as suggested by Cunnane et al., the body of autopsy research suffers from methodological factors [30] that limit the significance of these findings. The reliance upon autopsy studies to gather data on changes to brain DHA in disease states, and the inconsistences of these data, have turned researchers to look for peripheral markers of DHA status.

The DHA status of certain blood components has shown association with clinical markers of AD. Increased serum DHA is associated with reduced cerebral amyloidosis [60] and severity of AD [74], while greater plasma DHA is inversely correlated with the risk of developing AD over 10 years [75,76]. Similarly, greater erythrocyte DHA content corresponds with reduced risk of cognitive decline [77]. More recently, a meta-analysis has shown that altered blood fatty acid composition is evident in individuals with MCI and AD versus controls, with individuals with AD showing the greatest number of altered fatty acids and degree of change of these fatty acids, including DHA [78].

Dietary DHA intake can modify the DHA status of peripheral tissues such as blood and adipose tissue [79,80,81,82], dietary DHA correlates with hippocampal volume in vivo [83], and neurological deficits of DHA deficiency resolve with supplementation [84]. Together this suggests that dietary DHA supplementation may benefit brain DHA status, structure and function. As peripheral DHA status correlates with improved cognitive function and reduced AD risk, it would therefore be expected that these markers would correlate well with brain DHA status. One recent study provided cognitively normal, overweight individuals with either 2 g of DHA or soy placebo daily for 6 months [85]. In comparison to the soy control, DHA supplementation increased both plasma and cerebrospinal fluid (CSF) DHA status. While plasma DHA increased by 200%, CSF DHA increased by only 28%. This study indicates that dietary DHA crosses the blood–brain barrier (BBB) to enter CSF, but that the change in CSF DHA was marginal in comparison to that in plasma, and further reduced in individuals carrying the APOE4 gene. While encouraging, it is unknown whether CSF enrichment with DHA translates into integration of DHA into and functional activity within key brain structures affected in AD; this may relate to the failure of this study to show improvements in hippocampal volume or performance on cognitive assessments. With regard to studies assessing brain parenchymal DHA content, only one study has paired plasma and brain DHA values [86], while one other study linked subcutaneous adipose tissue and brain DHA status [87]. Both studies found no significant relationship between peripheral and brain DHA status in AD, demonstrating that although DHA may exert positive effects upon AD risk or pathophysiology, it is uncertain whether its mechanism of action is via increasing brain DHA content.

### Supplemental DHA Does Not Effectively Prevent or Treat AD

Despite the merits of DHA shown in epidemiological, pre-clinical, and in vivo studies, these successes have not been borne out in the prevention or treatment of cognitive decline and AD. No association between DHA in serum and clinical measures of cognitive performance (mini-mental state exam, MMSE) were found in baseline measurements from the OmegaAD trial, detracting from the belief in DHA’s effects on cognitive function [88]. Meta-analyses have found no benefit of N3 supplementation to prevent cognitive decline in cognitively normal elderly [89] or in the prevention and treatment of AD [90]. Post hoc analysis of cross-sectional studies by Cunnane et al. found no difference in blood DHA values between individuals with AD or cognitive decline and controls [91]. More recently, Alex et al. performed a systematic review of 25 studies assessing the effects of LCPUFA upon the cognitive performance of non-demented individuals of an average age of 57 years. This group found that supplementation with LCPUFA was associated with mild improvements in memory, however, with heterogeneity and asymmetry suggestive of publication bias. In agreement with historical systematic reviews, these analyses found no effect of LCPUFA upon specific cognitive domains such as visuospatial function or wider tests of global cognitive assessment [92]. In a similar meta-analysis, Zhang et al. [93] analyzed the effects of N3 PUFA supplementation upon cognitive function in individuals aged >65 years with established mild cognitive impairment (MCI). These authors concluded that LCPUFA may improve MMSE score in individuals with MCI, but that the results remain inconsistent. On the other hand, Balachandar et al. related the effects of DHA supplementation with age-related cognitive decline. In contrast to the findings of the previous two recent meta-analyses, no benefit to any cognitive domain including memory and executive function was found [94]. 

Epidemiologically, fish intake is associated with reduced AD risk, but studies of DHA in fish oil supplement form find little benefit to cognitive decline and dementia, despite effectively increasing DHA status in blood [95,96,97]. Similarly, Morris et al. found that one seafood meal per week reduced AD neuropathology, while DHA supplementation did not [58]. A broad meta-analysis assessing the effects of multiple dietary foods upon neurodegeneration found that while an inverse association between fish intake and neurodegenerative diseases was supported by moderate evidence, no association between AD and PUFAs was found, with low or very low quality of evidence [98].

Finally, another study found that dietary DHA increases DHA in plasma phospholipids and in cerebrospinal fluid, indicating successful penetration of the BBB, yet no significant differences in the rate of loss of cognitive function or brain volume were found versus placebo [99]. So while DHA has shown positive benefit in some studies, the heterogeneity of evidence in clinical trials remains mismatched against seemingly clear-cut epidemiological and pre-clinical evidence.

## 4. The Metabolic Fates of DHA Govern Uptake into Specific Tissues

**Figure 1:**
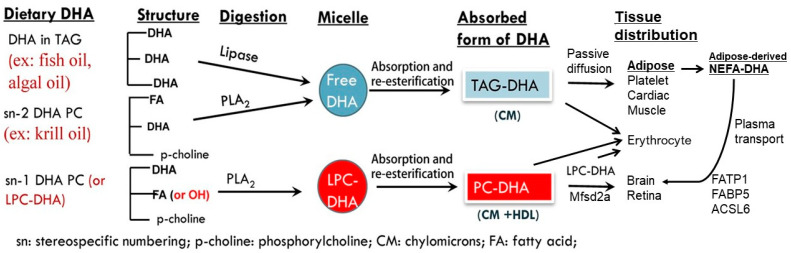
The metabolic fates of dietary DHA forms govern uptake into plasma lipid pools and influences tissue distribution. Of note is that lipolysis of DHA from adipose to produce NEFA provides a critical pool of long-term DHA supply for the brain. Figure modified from original published by Sugasini et al. (2017) [106].

Fish and other seafoods contain DHA esterified into TGs and PLs. In DHA supplements, such as fish oil capsules, DHA may be found as ethyl-esters, NEFA, and re-esterified TGs [100], but is rarely in PL form [31,101]. On the other hand, DHA supplements derived from krill oil contain DHA in NEFA, TG and PL forms [102], hence may more closely represent whole fish than does fish oil. Furthermore, some studies have prepared lysophosphatidylcholine (LPC) DHA for dietary ingestion by cleaving phospholipid-DHA (PL-DHA) using snake venom phospholipase [103,104,105]. One major area of discussion has been whether certain forms of DHA, particularly as PLs, are more likely to be taken up into the brain and to be more efficacious for prevention or treatment of AD [101]. This is because the different forms of DHA presented in food and supplements undergo divergent pathways of digestion and metabolism to differentially contribute to plasma lipid pools which in turn influence uptake into tissues. This is summarized in Figure 1. The following section describes the digestion and metabolism of dietary DHA forms into two main plasma lipid pools of lipoprotein-carried TGs and PLs and their subsequent availability to specific tissues.

### 4.1. Digestion and Metabolism of Dietary DHA

TG-DHA in seafood and fish oil supplements, and ethyl-ester-DHA as found in fish oil, are hydrolyzed by pancreatic lipase in the gut before the entry of their constituent fatty acids into enterocytes, re-esterification into TG-DHA, and transportation in chylomicrons to plasma via the lymphatic duct [106]. PL-DHA contained in fish and krill oil may hold DHA esterified at either the sn1 or sn2 positions. Dietary PLs are cleaved by enteric phospholipase at the sn2 position, releasing a NEFA and a lysophospholipid such as LPC [107]. Cleavage of dietary PL- DHA at the sn2 position forms a lysophospholipid and NEFA-DHA [106]. The latter is absorbed and re-esterified into TG-DHA in the enterocyte before entering the lymphatic duct to join the circulating plasma [108]. Conversely, if PL-DHA esterified at sn1 is provided, it may be cleaved to form LPC-DHA [109] and absorbed in that form or converted to PL-DHA in the enterocyte [32,110].

### 4.2. Metabolic Fates of TG- and PL-DHA

Dietary TG-DHA and PL-DHA enrich different plasma lipoprotein fractions with DHA. In broad terms, the metabolic fate of dietary PL-DHA and LPC-DHA is to be transferred from chylomicrons into high-density lipoproteins [109,111,112,113] (HDL) in the gut [109] and in the liver [112], while dietary TGs are readily incorporated into low density lipoproteins (LDL) [113] and very low density lipoproteins (VLDL) [114,115]. Thus, DHA forms derived from different dietary sources enter plasma bound to specific lipoprotein carriers, thereby influencing the availability and uptake of DHA into specific tissues. The metabolic fates of dietary DHA with respect to tissue distribution is summarized in Figure 1.

Although both are carried in plasma, TG-DHA and PL-DHA enrich different blood cells. Lemaitre-Delaunay et al. used radiolabeling techniques to show that although erythrocyte membranes are contributed to by NEFA, PL and LPC, PL-DHA is the main supplier [108]. On the other hand, the DHA supply of platelets is solely from NEFA-DHA derived from dietary TG-DHA sources [108,115]. Similar to erythrocytes, cardiac tissue receives DHA from all plasma sources [32,106]. The liver incorporates dietary phospholipid-DHA to a greater extent than dietary TG-DHA [116], while LPC-DHA may produce the greatest enrichment of hepatic tissue [106]. On the other hand, adipose tissue shows considerable uptake of TG-DHA from LDL and VLDL [117]. Sugasini et al. found that of LPC-DHA, PL-DHA and TG-DHA, only TG-DHA entered adipose stores [106], and Chouinard-Watkins et al. found TG-DHA enriches adipose ~3-fold more than does PL-DHA [118]. Indeed, PET-CT shows that the majority of plasma NEFA-DHA incorporates into peripheral tissues [119], and much of the TG-DHA content of chylomicrons is deposited in peripheral tissues such as adipose and muscle in the postprandial period [117], returning to the liver as depleted remnant chylomicrons.

Finally, the metabolic fate of dietary forms has been proposed to significantly affect brain DHA uptake. While TG-DHA disappears from circulation into adipose, HDL-bound PL-DHA persists in plasma and is metabolized into LPC-DHA by the liver and in plasma. LPC-DHA enters the brain via the Mfsd2a transporter. This metabolic fate of PL- and LPC-DHA has been proposed as the preferential mechanism of DHA brain uptake. Furthermore, this mechanism may explain why TG-rich/PL-poor fish oil supplements consistently fail to prevent or ameliorate cognitive decline and AD in clinical trials [106].

## 5. Plasma DHA Forms Supply Specific Tissues including Brain

**Figure 2:**
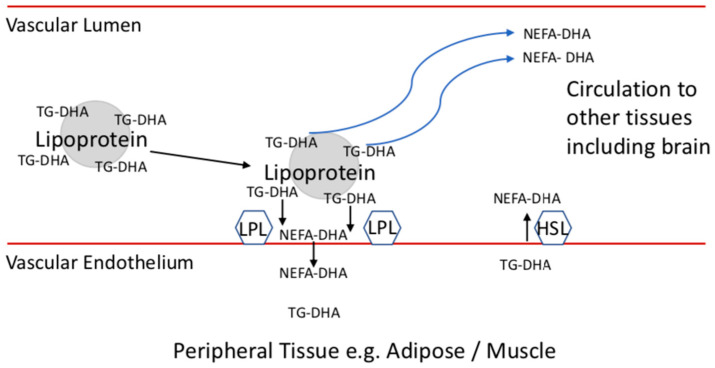
Regulation of fatty acid flux into and out of adipose tissue stores. A total of 50% of NEFA-DHA liberated by the lipolysis of TG-DHA at peripheral tissues by lipoprotein lipase (LPL) can escape into plasma, while NEFA are released by hormone sensitive lipase (HSL) during the fasting state [123]. These NEFA circulate elsewhere in the body to provide for other organs including brain [122]. Abbreviations: TG-DHA, DHA esterified to triglyceride; NEFA-DHA, non-esterified DHA.

Brain uptake of fatty acids and other substances is selectively controlled by the BBB. Lipoprotein-transported TG-DHA and PL-DHA do not directly supply brain via lipoprotein receptors, unlike other peripheral tissues [120,121], but lipoproteins can indirectly supply brain by transporting and liberating DHA to enter via alternative uptake mechanisms; 50% of NEFA-DHA liberated by the lipolysis of TG-DHA at peripheral tissues by lipoprotein lipase (LPL) can escape into plasma [122,123,124]. Radiolabeled palmitic acid shows preferential brain uptake in NEFA form, in comparison to its TG or cholesteryl ester forms in plasma [125]. Furthermore, a constant flux between plasma and adipose tissue in both fasting and postprandial states provides a constant contribution of plasma NEFA to tissues including brain [123,124] (Figure 2). Plasma NEFA-DHA crosses the blood–brain barrier via a ‘flip-flop’ mechanism facilitated by membranal Fatty Acid Transport Protein 1 (FATP1) [126], while diffusion gradients are maintained via FATP1, acyl-coA synthetase long-chain family member 6 (ACSL6) and Fatty Acid-Binding Protein 5 (FABP5) [127,128,129,130]. Defects in this mechanism result in impaired cortical DHA, neuroinflammation and reduced cognitive performance [127,131].

Lipoprotein-bound PL-DHA is metabolized into LPC-DHA in the plasma by Lecithin Cholesteryl Acyl Transferase (LCAT) [103,105], and by the endothelium-bound Endothelial Lipase (EL), found on the vascular endothelium of multiple tissues including brain [104,132,133,134]. LPC is also produced in the liver by Hepatic Lipase [135], of which ~5% is LPC-DHA [136]. LPC-DHA crosses the blood–brain barrier via the Mfsd2a transporter, deficiency of which is associated with microcephaly [35,137]. Thus, the dominant supplier of brain DHA is controversial. Supply can be fulfilled by DHA originating from plasma NEFA-DHA or TG-DHA; however, plasma PL-DHA and TG-DHA have also been suggested to be the dominant supplier of the brain [31,34,137,138].

The triad of PL-DHA, LPC-DHA and Mfsd2a as the main supplier of DHA to the brain has received strong support [31,33,36,106,139]. Studies have shown preferential uptake of LPC-DHA across in vitro BBB [140], privileged carriage of an LPC-DHA analogue into brain [141], after oral and intravenous administration of LPC-DHA [139,142], and after feeding LPC-DHA generated by lipase-altered krill oil [33], consistently elevating brain DHA to a greater extent and rate than TG-DHA [32,106].

It is therefore clear that the PL-DHA/LPC-DHA/Mfsd2a triad can increase brain DHA; however, the studies used to elucidate this pathway do not necessarily resemble normal physiological conditions. For example, LPC is not a significant constituent of a normal diet but is produced in small amounts in the gut and liver to achieve low physiological plasma concentrations of ~90 nmol/mL, with DHA making up only 1–5% of LPC-associated fatty acids [136,143]. Though further work to examine diet and physiological influences on plasma LPC is needed [34], studies that have shown preferential uptake into brain after administration of intravenous LPC-DHA, or oral LPC-DHA derived from enzymatically modified PL-DHA (via snake venom phospholipase), do not represent normal physiology but instead represent an augmentation of physiology with a synthetic dietary supplement (LPC-DHA), or bypass normal gut and hepatic metabolism completely using parenteral administration [104,105,106]. This should be held in mind both when considering claims that LPC-DHA via Mfsd2a-mediated transport is the major source of brain DHA, as well as when considering the potential benefit of therapeutic administration of LPC-DHA [36].

Much of the work showing dramatic increases in brain DHA status have been with provision of LPC-DHA, as described above. Therefore, direct comparison of oral PL-DHA and TG-DHA, as found in food or supplements, is more valuable to understand brain supply in the context of normal dietary intakes. Furthermore, these studies highlight an interesting relationship between dietary form and time as factors affecting brain DHA uptake. These studies are summarized in Table 1 listed in the appendices.

Short-term studies support preferential brain uptake of PL-DHA compared to TG-DHA; 10 week old rats showed 5–7-fold higher brain DHA accretion at 6 h after gavage with PL-DHA [118] and continued superiority to TG-DHA at 24 h [116]. Supplementation for 6 days showed increased uptake of PL-DHA into piglet brain, retina, liver and erythrocytes [146]. On the other hand, the same authors calculated the unlabeled DHA mass of brain tissue to conclude that the majority of DHA content and thus long-term supply of brain DHA was derived from dietary TG-DHA [146]. Similarly, Brossard found that after ingestion of radiolabeled TG-DHA, subsequently-produced radiolabeled LPC-DHA was the major supply to erythrocytes as compared to radiolabeled NEFA-DHA when studied over 80 h [155]. However, work by Vaisman providing 3 months of TG- or PL-DHA supplementation resulted in equal uptake into erythrocytes [151], suggesting an equalization of effect with time.

A number of studies designed to sample at both acute and delayed time points demonstrate changing dominance from PL-DHA to TG-DHA over time. Cook et al. found that at 12 h, PL-DHA from herring roe demonstrated an ‘area under the curve’ (AUC) in plasma phosphatidylcholine double that of TG-DHA from fish oil, indicating greater bioavailability from gut to tissues, yet plasma phosphatidylcholine levels of DHA were not significantly different between groups at two weeks [152]. Kitson et al. found that 6 h after an oral bolus of PL-DHA or TG-DHA, rat hippocampal uptake was 140% greater in the PL group as compared to TG-DHA. After 2 weeks of daily administration of PL-DHA, TG-DHA, or a TG-/PL-DHA mixture, however, no significant differences were found between groups. While the TG-/PL-DHA mixture was not studied over 6 h, its ability after 2 weeks to equally supply DHA as compared to TG- and PL-DHA alone further suggests that time, regardless of form, facilitates maximal brain uptake [154].

Sampling after 4 weeks, Yurko-Mauro et al. found similar plasma and erythrocyte bioavailability between krill oil and fish oil, which are enriched in PL-DHA or TG-DHA, respectively [147]. Similarly, PL- or TG-DHA given to mice for 5 weeks found no difference to DHA concentration in adipose, liver or brain [148]. Furthermore, no difference in brain uptake between PL- and TG_DHA forms was found by Ahn et al. at 2 weeks [153] or Hiratsuka et al. at 4 weeks [149]. Studying DHA accretion into rat peripheral tissues over 6 weeks, no difference between krill oil or fish oil was found [150]. Other studies of 72 h duration and greater have found absent or non-significant differences between PL- and TG-DHA as krill oil, krill meal or fish oil with regard to incorporation into plasma phospholipids [144,145]. These results show that, while the PL form provides rapid elevation of plasma and brain DHA shortly after administration, passing time allows the effects of dietary TG-DHA to equalize and even surpass PL-DHA to be the major supplier of tissue and brain DHA. This conclusion is in agreement with synthesis by Bazinet et al. [34], who proposed that circulating LPC-DHA has a higher brain/body partition coefficient, providing more DHA to the brain per unit of LPC-DHA, but that NEFA-DHA has a greater net entry into the brain and is the main supplier. The idea that tissue uptake is more dependent on time than the dietary form complements the kinetic studies of Chen et al. [156], demonstrating that LPC-DHA rapidly incorporates into plasma and brain to acutely provide 3-fold more DHA to brain than TG-DHA, but that when calculated relative to plasma half-life, TG-DHA liberated into NEFA supplies 10-fold more DHA to brain. Indeed, the rate of NEFA-DHA uptake matches the rate of DHA consumption of the brain, while accretion rates of LPC-DHA exceeds it, leading to authors arguing that NEFA-DHA is the sole source necessary for brain DHA homeostasis [9,157].

The mechanisms by which dietary TG-DHA provides a sustained and superior source of DHA to the brain are unknown, but as brain lipoprotein receptors do not uptake lipoprotein-bound TGs, dietary TG-DHA cannot be taken up into the brain and must be converted to plasma NEFA-DHA instead. The extended temporal nature of DHA accretion into brain makes dietary DHA a less likely candidate to be the main supplier of brain DHA. It is more likely to be explained by the storage of dietary DHA first in adipose tissue, whereby subsequent lipolysis provides a source of DHA at a rate that matches brain DHA flux and mitigates dependence on regular dietary DHA intake, as outlined below.

## 6. Adipose Stores and Supplies NEFA-DHA to Tissues including Brain

Multiple studies show preferential deposition of LDL- and VLDL-transported TG-DHA into adipose [106,117,118]. Similarly, the major fates of the N3 PUFA alpha-linolenic acid, precursor to DHA, are beta-oxidation or sequestration into adipose tissue [11]. The specific tissue partitioning of PL-DHA and TG-DHA may be an oversimplification as studies in mice found both PL-DHA and TG-DHA contribute to adipose DHA in a dose-dependent manner [148]. While studies focus on the ability of LPC-DHA to supply brain in the short term and generally disregard TG-DHA, the preferential uptake of dietary TG-DHA into adipose may represent its superiority as a DHA source for the brain in the long-term. Fatty acids stored in adipose may have a half-life of 1–2 years [158] and are stable with age [159]. Despite its stability, both feeding and fasting stimulate the actions of LPL and hormone sensitive lipase, respectively, creating a constant flux of diet- and adipose-derived NEFA released into plasma [123,124] (Figure 2).

In contrast to plasma TGs and PLs that contain 0.8% and 3.6% of total fatty acids as DHA, respectively, subcutaneous adipose tissue contains 0.2% of its fatty acids as DHA [160]. Despite adipose containing DHA at a lower percentage of total fatty acids, Western humans hold 26–30% of their total body weight as non-essential adipose tissue, thus providing a large mobilizable source of fatty acids including DHA [161]. In total, adult adipose stores contain 50 g of DHA [161], an amount calculated to be adequate to supply the brain for 14–36 years [11,156]. Chen et al. estimated plasma NEFA-DHA flux to fully meet the demands of brain DHA turnover based on their calculations, which is in agreement with those of Umhau et al. [11,119,156]

Further attempts to accurately measure the contribution of dietary TG-DHA and plasma NEFA-DHA to brain DHA homeostasis may assess the interrelationship of dietary DHA, adipose-derived NEFA-DHA, and brain DHA uptake. Indeed, the storage and supply function of adipose is essential during neurological development in utero and in the postnatal period. DHA accrual in utero increases exponentially in the third trimester [162], paired with growth of both brain and adipose stores. At term, adipose holds 7-fold more DHA than does brain [163] and receives 90% of maternally-derived energy [164]. After birth, DHA stored in fetal adipose tissue is utilized for tissue growth [162,165]. If fed DHA-deficient formula, infant DHA stores in adipose reduce to unmeasurable levels due to their consumption to provide DHA for postnatal brain development [166] and DHA deficiency may contribute to lower IQ scores in formula-fed infants compared to breast-fed infants [14], demonstrating that adipose tissue provides an essential supply of DHA to fulfil requirements during periods of high demand. The relationship between mother and fetus demonstrates that adipose provides a mobilizable and vital source of DHA for the brain. Mothers provide 42 mg of DHA per day via placental transfer to the fetus during the final 5 weeks of pregnancy, providing DHA largely derived from maternal adipose stores even in circumstances of maternal DHA deficiency, in a process termed ‘biomagnification’ [167,168]. During breastfeeding, LCPUFA are derived from synthesis and diet but mainly from the maternal adipose tissue, providing around 60 mg of DHA per day [161]. The adipose stores of DHA-replete lactating mothers decrease during the breastfeeding period, while mothers who are DHA-deplete are unable to meet the DHA demands of the feeding infant [161].

The dependency of fetal brain growth upon maternal mobilization of DHA stored in adipose both in utero and during lactation, followed by postnatal reliance of infant adipose DHA stores to continue supplying the brain, demonstrates physiological extremes that show how adipose may provide a significant depot in the event of DHA demand in the adult. In this scenario, adipose DHA stores would vastly exceed the amounts needed to meet the adult brain’s daily DHA consumption rate [119]. While plausible, the ability of non-pregnant adult adipose to release stored DHA to other tissues such as brain has not yet been characterized, though the authors feel that adipose tissue should be considered an active sink of DHA in any study assessing the bioavailability and distribution of supplemental LCPUFA. 

## 7. Adipose Tissue Is an Unknown Entity in Alzheimer’s Disease

If associations between AD and reduced DHA in plasma, erythrocyte and brain are correct, consumption of adipose DHA stores may be expected to be increased in AD. Only one study has studied adipose DHA in AD, having performed pairwise comparisons of adipose DHA between AD individuals and their cohabiting proxies who were assumed to share similar dietary intake of DHA, but found no significant difference [87]. Therefore, if normal physiological conditions allow dietary and adipose sources of NEFA-DHA to be the main source of DHA, but fish oil supplementation is ineffective for AD treatment and adipose DHA is unaffected in individuals with AD, where is the source of the mismatch?

## 8. AD Risk Factors Alter DHA Homeostasis

Increasing age, the APOE4 genotype, and cardio-metabolic disease act independently and synergistically to increase the risk of AD development [4]. Furthermore, they may act to disrupt DHA homeostasis by affecting synthesis, metabolism and uptake into brain.

### 8.1. Age and Metabolic Disease

Aging-related decreases in hepatic desaturase and elongase enzymes may reduce DHA synthesis from N3 PUFA precursors [169], increasing reliance on DHA-replete diets. Ageing is associated with abnormal plasma lipid metabolism [170] such as prolonged and higher elevations in DHA after oral fish oil than in younger counterparts, accompanied by higher erythrocyte and plasma DHA levels [170] and increased beta-oxidation and retroconversion [169]. Persistence within plasma suggests that DHA does not enter peripheral tissues and must be disposed of by alternate means of metabolism. Ageing is also associated with increasing insulin resistance [171] and Type 2 Diabetes (T2D) is associated with 60–65% greater risk of developing AD [172,173,174]. If developed in mid-life, T2D is associated with brain glucose hypometabolism, amyloid accumulation and progressive neurodegeneration [175,176,177]. Overcoming brain insulin resistance in AD patients by administering insulin infusions improves cognition [178].

Despite plasma hyperinsulinemia, AD brain demonstrates reduced endothelial insulin receptors and decreased insulin in cerebrospinal fluid (CSF) [179,180] suggesting reduced transport of insulin into brain, where insulin has myriad effects on all brain cell types including anti-amyloid activity [181]. Insulin stimulates DHA uptake via FATP1 into endothelial cells and acyl-coA-synthetase activity of FATP4 in muscle [126,182], suggesting that insulin-mediated transport of PUFA into brain is possible. Age-related insulin resistance could impair plasma DHA uptake into brain and peripheral tissues, resulting in the elevated plasma DHA seen by Plourde and Vandal despite having a relatively greater DHA plasma [183,184]. Thus, in insulin resistant states, mechanisms to bypass FATP1 mediated passive diffusion by utilizing the Mfsd2a transporter may be of benefit.

### 8.2. APOE4 and the BBB

Apolipoprotein E4 (APOE4) is the most important genetic risk factor for late-onset AD [185] and is amplified by other factors including age, gender [186], smoking and physical inactivity [3]. APOE4 carrier status is associated with lipid metabolism abnormalities [187,188,189] including reduced incorporation of dietary N3 PUFA into plasma free fatty acids and TGs [190], greater beta-oxidation of DHA and a reduced whole-body DHA half-life [191], changes expected to reduce plasma DHA availability to target tissues. Individuals carrying the APOE4 genotype have been noted to have reduced or absent benefits to AD risk derived from fish intake [192]. As described recently, APOE4 disrupts the BBB and brain DHA homeostasis, but this pathology may be amenable to treatment with PL-DHA or LPC-DHA via the Mfsd2a transporter [101]. Abnormal BBB physiology is also a feature shared with ageing [193], hyperinsulinemia and hyperglycemia [181,193]. It is clear that in these four key risk factors for AD, followed by disruptions of DHA homeostasis and uptake into brain, may contribute to resistance to NEFA-DHA provided by diet, fish oil supplements and adipose, hence providing an opportunity to circumvent these barriers via alternative DHA forms such as LPC-DHA via the Mfsd2a transporter [34,101].

## 9. Is There an Optimal Level of Brain DHA?

When defining the level of appropriate delivery of DHA to the brain, we must consider that there may be a ceiling above which DHA concentration in the brain becomes excessive. Although this level is unknown, it may be inferred by the findings of retroconversion of DHA into other N3 fatty acids via an ‘entropically and energetically expensive’ process seen in studies showing greater brain uptake of PL-DHA as compared to TG-DHA [146,154]. Similar processes occur to maintain low brain levels of other N3 PUFAs, such as eicosapentaenoic acid (EPA) [194]. Beta-oxidation and retroversion of DHA also occurs in the plasma of elderly humans, following impaired metabolism and tissue-uptake of a DHA bolus [191]. By contrast, during developmental periods, retroconversion of DHA in brain occurs in only minor amounts [195], reflecting high demand for and utilization of DHA.

If a ceiling for brain DHA were to exist, it would for good reason. AD pathogenesis includes mitochondrial dysfunction and excessive oxidative stress [196,197], which may expose DHA to free radicals and lipid peroxidation. Lipid peroxidation can produce reactive aldehydes such as acrolein and 4-hydroxynonenal (4-HNE) that contribute to NFTs, pathogenic hallmarks of AD [198]. Peroxidized lipids form further reactive species that propagate lipid peroxidation and ongoing membrane damage [199]. DHA is highly concentrated in phospholipid membranes to aid synaptic neurotransmission [200] and is also highly susceptible to peroxidation [201,202,203]. In retinal tissue, where DHA is highly concentrated, greater DHA levels associate with greater oxidative damage [204]. In the case of oxidative pathology in the brain, excesses of DHA would therefore be assumed to be undesirable. Importantly, we must not to confound the physiological effects of DHA with the physical nature of the DHA molecule. While DHA certainly has anti-inflammatory and anti-oxidant effects, it is still prone to oxidation by free radicals if not adequately protected or present in excess. For instance, studies of the effects of oxidized PUFA in human neuroblastoma and mouse cortical cell lines showed that only minor amounts (1%) of oxidized DHA reverts the protective effects of DHA and increases amyloidogenic processing of amyloid precursor protein to amyloid beta [205]. Therefore, if therapeutic doses of DHA in PL or LPC form are provided to those at risk of AD (e.g., in the setting of insulin resistance or APOE4 carriers), we must also be wary of the possibility of providing excess DHA, which may exacerbate oxidative injury in the at-risk brain.

### Where to from Here?

This paper discusses the metabolic fates of dietary forms and sources of DHA with respect to tissue uptake. Although different forms appear to have defined metabolic fates in the short term, this relationship dissipates with increasing time, with equivalence of delivery to the brain beginning around 3 days after commencing supplementation when normal brain DHA uptake mechanisms are intact.

This interaction between dietary form, time duration, and adipose tissue function is something that has not been fully considered in the research assessing DHA homeostasis in Alzheimer’s Disease, but has implications for the interpretation of research already completed and for the planning of future work.

Previous clinical trials have failed to show consistent benefit to the prevention and amelioration of cognitive decline and AD. The reason for these failures is unknown, but has been suggested to be influenced by the metabolic fate of TG-rich fish oil supplementation, thus proposing that a solution may be found in the provision of PL-rich sources of DHA such as in krill oil, or in LPC-rich preparations. Indeed, there has been a call for clinical trials evaluating the effects of PL-DHA in individuals with APOE4 and AD [101]. Solely manipulating dietary form to increase brain DHA with the hope of improving cognitive health status, however, neglects the added dimension of time and the substantial influence of adipose tissue upon plasma DHA availability to organs such as brain. Before studies of exogenous DHA from supplements are started, we must better understand the flux of DHA between diet, plasma and adipose and the contribution of adipose-DHA to brain DHA homeostasis.

We suggest that dietary PL-DHA directly enters the brain in the short term, but TG-DHA indirectly supplies the brain after being held in the substantial DHA stores of adipose tissue. In this way, the depot and supply function of adipose negates fluctuations in dietary supply, as evidenced in neonatal development, to provide a steady source of DHA to the brain. In fact, the adult body already contains ample DHA, as new mothers can mobilize 50 g of DHA from their adipose for their developing infant [161]. In this scenario, it is apparent that adipose tissue health and function is more important than the form of dietary DHA. It may be that DHA deficiency in AD is not solely a product of dietary DHA deficiency, but further aggravated by metabolic dysfunction affecting adipose tissue and plasma lipid pools. 

Furthermore, much work has been done to relate dietary intake of DHA and PUFA intake with measurements of peripheral tissues such as adipose, erythrocyte membrane, serum and plasma, in order to establish a biomarker of DHA status [206]. For example, the DHA content of erythrocyte membranes represents exposure to DHA in the past 180 days [207] and has been used as a marker of dietary DHA intake. The identification of biomarkers of DHA status therefore focuses on diet and does not acknowledge the supply function of adipose tissue. Even in the absence of dietary DHA, stores of adipose DHA released into plasma will influence the DHA content of plasma and erythrocytes, as shown by NEFA, PL and LPC contributing to erythrocyte membranes [108], or after hepatic production of LPC from adipose-derived NEFA [115]. Indeed, correlations between diet and erythrocytes suffer from high variability [208], which may be explained by inter-individual differences in adipose tissue DHA storage and release. A greater understanding of the influence of adipose tissue upon peripheral markers of DHA status and that of brain may further help to understand why attempts to establish the DHA content of plasma and adipose as corollaries of brain DHA have been unsuccessful [86,87].

The unknown influence of adipose tissue may also affect the validity of clinically relevant blood biomarkers of N6 PUFA status. The omega-3 index, the percentage of EPA plus DHA in erythrocytes, has been used to determine aspects of cardiovascular disease risk [209,210,211]. A target omega-3 index value of erythrocytes has been suggested to be optimal and is sought by increasing dietary intake of DHA via food or supplements or potentially by reducing dietary N6 PUFAs. However, due to the metabolic fate of TG-DHA, any increase in erythrocyte DHA is likely accompanied by a greater distribution of ingested DHA into adipose stores. Adipose inflammation is a known contributor to cardiovascular disease [212], but DHA and N3 PUFAs act to decrease adipose inflammation to improve metabolic health [213,214,215,216]. Thus, a favorable omega-3 index value of erythrocytes may be a proxy marker of cardiovascular benefit due to modulation of adipose tissue function.

The role of adipose tissue in plasma and tissue DHA homeostasis is made more complex by its depot and supply of other fatty acids. One example is of N6 PUFAs, such as linoleic acid (LA) and arachidonic acid (AA), whose presence in the diet interacts with dietary N3 PUFA to impair N3 PUFA synthesis and reduce levels of N3 PUFA in tissues [217,218,219]. If, like DHA, the distribution of N6 PUFA is influenced by both dietary and adipose sources, any trial attempting to improve DHA status by reducing dietary N6 PUFAs must also consider pre-ingested dietary N6 PUFA stored in adipose. Furthermore, oxidation products of N6 PUFAs such as LA are linked to pathologies including cardiovascular disease and AD, and can be reduced by dietary N6 PUFA restriction [219,220]. While SPMs produced via the lipoxygenation of DHA are generally thought to have positive effects [221], lipid mediators derived from oxygenated N6 PUFA are often associated with pro-inflammatory effects [222] and may contribute to chronic inflammation in the absence of counterbalanced SPMs derived from N3 PUFA. Thus, the benefits of DHA supplementation upon adipose tissue inflammation may in part be brought about via reductions in adipose N6 PUFA content or effect. 

AD has multiple pathological mechanisms that may disrupt the ability of the relationship between diet, adipose and time to provide DHA to the brain. As discussed, alterations to BBB transport due to age, APOE4 status or endothelial dysfunction may limit uptake of NEFA, providing a sole route of potential DHA transport via the Mfsd2a transporter. Peripheral insulin resistance and metabolic disease may alter plasma lipid transport and adipose function, preventing flux of DHA between plasma, adipose and brain. In these circumstances, forms of DHA with metabolic fates designed to bypass altered DHA homeostasis may be useful clinical tools in scenarios of chronic or acute neurological disease [36,223,224]. These forms may include PL-DHA as found in food, enzymatically-modified DHA containing lysophospholipids, or as synthetic LPC analogues such as 1-acetyl,2-docoshexaenoyl-glycerophosphocholine ‘AceDoPC’^®^ [141]. Before clinical application of these DHA forms can become reality, however, further work is necessary to understand optimal therapeutic parameters to avoid the risk of worsened oxidative pathology.

## 10. Conclusions

This paper has aimed to expand discussion surrounding the utilization of the metabolic fates of dietary and plasma forms of DHA to target tissue and brain uptake in both normal and diseased physiology. The form and source of DHA may influence tissue distribution in the short term, but long-term DHA homeostasis is more significantly influenced by the depot and supply function of adipose tissue. Dietary and plasma PL-DHA and LPC-DHA can rapidly increase brain DHA, but NEFA-DHA released from adipose tissue and from the diet provides the primary source of DHA for brain in normal physiology. Adipose represents a rich and dynamic source of DHA but its influence upon brain DHA status and neurological disease is not understood. Moving forwards, models assessing the relationship between dietary, plasma, adipose and brain DHA are needed, as well as a better understanding of how or whether peripheral measures of DHA status correlate with brain DHA content. Exploitation of the preferential uptake of PL-DHA and LPC-DHA may have clinical utility in disease states where DHA homeostasis is altered, but the potential risk of oversupply must also be considered. Therefore, if alternative forms of DHA are utilized therapeutically, we must better understand any potential negative effects of excessive DHA on AD neuropathology, in addition to its promise as a neurotherapeutic. 

## Figures and Tables

**Table 1:** Outcomes of studies comparing the abilities of DHA esterified to PL or TG to enrich tissues including brain. Studies are listed in order of duration of study. An arbitrary line drawn on the table at 72 h indicates an apparent shift of dominance of PL-DHA as the main supplier of DHA to tissues to greater equality of effect between PL- and TG-DHA with increasing length of supplementation. A subsection at the base of the table demonstrates two studies assessing both acute and delayed effects of PL- and TG-DHA supplementation. Abbreviations: DHA: Docosahexaenoic Acid, EPA: Eicosapentaenoic acid, FO: Fish oil, KO: Krill Oil, PL: Phospholipid, TG: Triglyceride, LPC: lysophosphatidylcholine, AUC: Area Under the Curve. In studies comparing KO vs. FO, KO is rich in PL-DHA and FO rich in TG-DHA.

Author	Model/Population	Tissue	Study Duration	Comparison	Results	Comment
Chouinard-Watkins et al., 2019 [118]	Rats	Brain cortex and serum lipid classes	6 h	DHA esterified to phosphatidylcholine (DHA-PtdCho), phosphatidylethanolamine (DHA-PtdEtn), phosphatidylserine (DHA-PtdSer) or as triglyceride (TG-DHA)	Brain DHA levels 5-7 fold higher in DHA-PtdCho and DHA-PtdSer groups than in TG-DHA group.	
Graf et al., 2010 [116]	Rats	Brain	24 h	PL- vs. TG-DHA	In 10-week-old rats, tissues such as liver, brain, kidney and anterior uveal tract (retina) accumulated 2–3 fold more PL-DHA-derived radioactivity than compared with TG-DHA. 14C-DHA derived radioactivity after 14C-DHA-PC dosing compared with 14C-DHA-TG dosing.	DHA-ester type did not influence tissue uptake in rats aged <10 weeks old, while age influenced tissue uptake regardless of DHA-ester type.
Köhler et al., 2015 [144]	Adult humans	Plasma phospholipids	72 h	Two krill products (krill oil, krill meal) vs. FO	A larger AUC of plasma DHA was detected for krill oil in comparison to krill meal or fish oil. Bioavailability of EPA+DHA was not different between krill meal and fish oil.	A large inter-individual variability in response was observed.
Schuchdart et al., 2011 [145]	Adult humans	Plasma phospholipids	72 h	Two FO products containing DHA as either ethyl-ester or re-esterified TG compared against KO	Nonsignificant differences in AUC and maximum plasma phospholipid concentration of DHA between all groups.	High standard deviation values.
Liu et al., 2014 [146]	Piglets	Brain	6 days	PL- vs. TG-DHA. Results normalised as %Dose of radiolabelled DHA found in the grey matter of the cerebral cortex for each PL-DHA and TG-DHA.	The %dose of PL-DHA was 1.9× more efficacious for grey matter DHA accretion than TG-DHA.	Less retro-conversion to N3 DPA in the TG-DHA group (PC > TG 2.8 fold). TG-DHA provided as 4.8 mg/500 mL feed vs. PL-DHA 1.8 mg/500 mL feed. Data regarding brain updake from total dietary TG-DHA/PL-DHA was not displayed.
Yurko-Mauro et al., 2015 [147]	Adult humans	Plasma and Erythrocyte	28 days	FO (containing DHA as either ethyl-ester or re-esterified TG) and KO.	No significant differences in plasma or erythrocyte EPA + DHA at 28 days between groups.	
Adkins et al., 2019 [148]	Mice	Liver, Adipose, Heart, Eye, Brain.	38 days	PL- vs. TG-DHA	No difference in brain DHA concentration.	
Hiratsuka et al., 2009 [149]	Mice	Liver and Brain	5 weeks	PL- vs. TG-DHA	No significant differences in brain or liver fatty acid contents or of DHA content.	
Ghasemifard et al., 2015 [150]	Rats	Whole body, Liver, heart, white gastrocnemius muscle and perirenal adipose tissue	6 weeks	FO vs. KO	No significant effect of diet on net accumulation of DHA.	
Vaisman et al., 2008 [151]	Children aged 8-13 years	Blood lipid profile	3 months	PL-DHA vs. FO	No significant change to blood lipids after three months.	
**Studies with acute and delayed measurement phases**						
**Author**	**Model/Population**	**Tissue**	**Study Duration**	**Comparison**	**First Timepoint Results**	**Second Timepoint Results**
Cook et al., 2016 [152]	Adult humans	Plasma phospholipids	12 h and 14 days	PL-rich herring roe oil or TG-rich FO	After 12 h, the ability of PL-DHA to increase the AUC of EPA, DHA and EPA +DHA was 2-fold that of TG-DHA.	After 2 weeks, there was no significant difference in the abilities of each supplement to increase plasma EPA+DHA.
Ahn et al., 2018 [153]	Rats	Blood and Brain	48 h and 14 days	FO and two forms of KO	TG-DHA increased brain DHA the most at 2 h, but PL-DHA in KO achieved greatest brain DHA at 48 h. The statistical significance of these findings was not described in the paper.	No significant difference in DHA content between FO, KO, and CKO groups in brain or blood.
Kitson et al., 2016 [154]	Rats	Brain	6 h and 4 weeks	PL- vs. TG-DHA	PL-DHA achieved 78%, 140% and 69% greater labelling in cerebellum, hippocampus and remainder of brain than the TG-DHA group.	No difference in brain DHA concentration between groups fed PC-DHA, TG-DHA or a combination of both PC- and TG-DHA.
